# First experimentally characterised *Proteus* phage endolysins have distinct metal cofactor dependencies

**DOI:** 10.1007/s00253-026-13891-1

**Published:** 2026-06-06

**Authors:** Akash Shambharkar, Thomas P. Thompson, Laura A. McClenaghan, Katie J. J. Brown, Stephen A. Kelly, Brendan F. Gilmore, Timofey Skvortsov

**Affiliations:** 1 School of Pharmacy, Queen’s University Belfast, Medical Biology Centre, 97 Lisburn Road, Belfast, BT9 7BL UK; 2 School of Medicine and Centre for Interventions in Infection, Inflammation, and Immunity (4i), University of Limerick, Limerick, Ireland

**Keywords:** Bacteriophage, Phage, Endolysin, *Proteus*, CHAP, Peptidase M15

## Abstract

**Abstract:**

*Proteus mirabilis* is a Gram-negative uropathogenic bacterial species responsible for many catheter-associated urinary tract infections (CAUTIs). Due to the growing rates of antimicrobial resistance among CAUTI pathogens, novel antimicrobial solutions are urgently needed. Bacteriophage endolysins are an emerging class of antimicrobial agents, but no endolysins from *P. mirabilis* phages have been reported to date. Here, we describe two new *Proteus* phage endolysins, LysPM1 and LysPM2, containing Peptidase_M15_3 and CHAP domains, respectively. We experimentally confirmed their antibacterial activity against several *Proteus* spp. when used in combination with outer membrane permeabilizers, including EDTA, citric acid, and chloroform, as well as against frozen bacteria. Both enzymes have optimum activity at pH 7–8 and retain activity at temperatures up to 60 °C. Both endolysins completely lost their lytic activity upon treatment with EDTA, suggesting that divalent cations participate in the catalytic mechanism; the addition of Ca2⁺ and Zn2⁺ to LysPM1, and Ca2⁺ and Mn2⁺ to LysPM2 partially restored the activity. Our study confirms LysPM1 and LysPM2 as the first experimentally characterized *Proteus* phage endolysins, with LysPM2 also being the first experimentally characterized Gram-negative endolysin with a single CHAP domain. These findings could guide further development of phage-derived lytic enzymes for treatment of *Proteus* infections.

**Graphical Abstract:**

**Figure:**
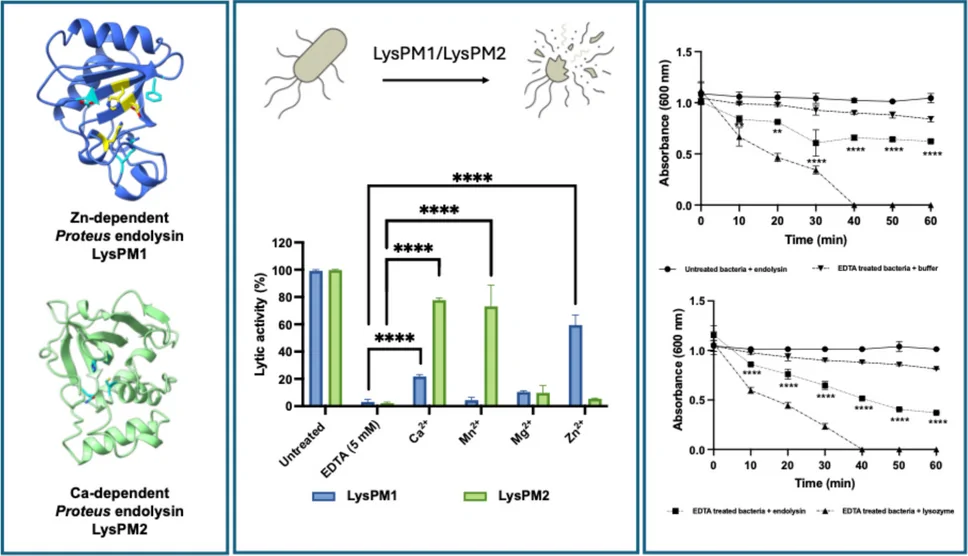

**Key points:**

• *LysPM1 & LysPM2 are the first experimentally characterized Proteus phage endolysins.*

• *Both are single-domain endolysins: Peptidase_M15_3 in LysPM1 and CHAP in LysPM2.*

• *Metal cofactors are required for their activity: Zn2⁺ for LysPM1, Ca2⁺ for LysPM2.*

**Supplementary Information:**

The online version contains supplementary material available at 10.1007/s00253-026-13891-1.

## Introduction

Antimicrobial resistance (AMR) and the spread of multidrug-resistant (MDR) bacteria are a major concern, posing a significant threat to public health worldwide (Naghavi et al. 2024; Salam et al. 2023). Among these pathogens, *Proteus mirabilis* is particularly notable for its significant impact on catheter-associated urinary tract infections (CAUTI) (Wasfi et al. 2020). *P. mirabilis* is an opportunistic Gram-negative pathogen, commonly linked to urinary tract infections in patients with anatomical or functional problems. This is particularly problematic in long-term catheterized patients (Wasfi et al. 2020). *P. mirabilis* produces the enzyme urease, elevating urinary pH, and leading to the accumulation of crystalline deposits on catheter surfaces. This can cause damage to the urethra and bladder epithelia, along with catheter blockage and urinary retention, increasing the risk of urinary tract infections and sepsis (Gmiter and Kaca 2022; Herout et al. 2023; Scavone et al. 2023). According to a number of recent reports, antibiotic resistance in *Proteus* spp. is on the rise (Facciolà et al. 2022; Hassuna et al. 2025; Zhang et al. 2025), necessitating the search for alternative options to traditional antibiotic therapy.

Bacteriophages are viruses that infect bacteria and propagate within them (Elois et al. 2023). Phages have been recognized as a promising alternative to antibiotics, with applications across medicine, the cosmetic industry, and the agri-food sector (Harper et al. 2021). In recent years, phage-derived enzymes, including endolysins, virion-associated peptidoglycan hydrolases (VAPGH), depolymerases, holins, and spanins have been actively investigated for their antibacterial and antibiofilm properties (Abeysekera et al. 2022; Aslam et al. 2021; Gontijo et al. 2021; Murray et al. 2021; Roach and Donovan 2015; Rodríguez-Rubio et al. 2013; Wang et al. 2024).

Endolysins (also termed lysins) have been shown to have substantial potential as an alternative solution to antibiotics for bacterial infection control. Endolysins are enzymes produced by the phage-infected cells during the final stage of the phage lytic cycle (Abdelrahman et al. 2021; Liu et al. 2023). They degrade the bacterial cell wall, helping to release newly formed phage particles, effectively killing the bacterial host cell (Oliveira et al. 2013). While phages represent a promising therapeutic option on their own, endolysins offer several advantages over whole phage therapy. Endolysins typically have a broader host range than their phages (Nakonieczna et al. 2015). Compared to phages, endolysins have faster bactericidal activity and are characterised by their non-proliferative, proteinaceous nature, which makes it easier to control and monitor local and systemic concentrations of endolysins, and prevents occurrence of spontaneous mutations (Liu et al. 2023). The modular structure of endolysins makes them amenable to genetic engineering, allowing the development of endolysins with two or more simultaneous lytic activities (Gerstmans et al. 2018; Nelson et al. 2012). Finally, while the emergence of resistance to endolysins is possible, it is a rare occurrence and should be considered of low concern due to the high specificity and rapid action of endolysins as well as the highly conserved structure of the peptidoglycan layer targeted by endolysins, making resistance development unlikely in real-life applications (Dams and Briers 2019). Endolysins thus represent a valuable addition to the list of antimicrobial options against antibiotic-resistant pathogens.

Here, we describe two novel endolysins isolated from *Proteus* phages. To the best of our knowledge, few endolysins have been demonstrated experimentally to have activity against *Proteus,* and none have previously been isolated from *Proteus mirabilis* phages. Given their demonstrated specificity and antibacterial activity against *Proteus,* our study expands the arsenal of tools available to treat *Proteus* infections.

## Experimental procedures

### Bacteriophages and bacterial strains used in this study

Phage vB_PmiS_PM-CJR (PM-CJR 'hereinafter') was previously isolated and characterized (Rice et al. 2021). Phage vB_PmiP-Homer (Homer 'hereinafter') was purchased from the German Collection of Microorganisms and Cell cultures GmbH (DSMZ DSM 107146; unpublished). The bacterial strains *P. mirabilis* BB2000 and *P. mirabilis* Hauser 1885 were used throughout the study for propagation of phages PM-CJR and Homer, respectively, as well as for the experimental characterization of phage endolysins. A full list of bacterial strains used in this study for host range testing of phages and their endolysins is provided in Table S1. All bacterial strains were cultured in lysogeny broth (LB) (Invitrogen, Paisley, UK) at 37 °C with shaking and maintained on low swarm agar (Invitrogen, Paisley, UK).

### Bioinformatic analysis of putative endolysins LysPM1 and LysPM2

DSMZ provided a nucleotide FASTA file containing the genomic sequences of Homer and a GenBank file with gene annotations. The gene encoding Homer phage endolysin was identified in the annotated GenBank file provided by DSMZ and its protein product was named LysPM1. We previously sequenced and annotated phage PM-CJR (GenBank accession no.: MZ643249.1). The protein encoded by the gene of the PM-CJR phage endolysin (vBPmiSPMCJR_062) was named LysPM2 (GenBank accession no. QYC51792). The protein sequences of both endolysins are provided in Table S2.

The molecular weights of LysPM1 and LysPM2 were predicted using an online version of the Protein Molecular Weight calculator available at Bioinformatics.org (Stothard 2000). Secondary structure analysis was performed with PSIPRED (Buchan et al. 2024). The tertiary structures of LysPM1 and LysPM2 were predicted using the AlphaFold 3 online server (Abramson et al. 2024) and visualized with ChimeraX (Meng et al. 2023). DALI and Foldseek servers were used for structural comparison of AlphaFold models of LysPM1 and LysPM2 to experimentally determined protein structures in the PDB (Holm 2022; van Kempen et al. 2024; wwPDB consortium 2018).

The endolysin sequences were analyzed using HHPred with default databases and parameters (Soding et al. 2005). InterProScan analysis with default parameters was used for domain prediction and functional characterization of the endolysins (Paysan-Lafosse et al. 2023).

The protein sequences of LysPM1 and LysPM2 endolysins were used as queries for a search with NCBI BlastP (Camacho et al. 2009) against the NCBI RefSeq protein database (refseq_protein) limiting the search to Viruses (taxid:10,239) with the default parameters. The search results were filtered to retain sequences with query coverage between 80 and 100. In addition, the sequences of LysPM1 and LysPM2 were searched against the MEROPS-MP database (Rawlings et al. 2018) using the EMBL-EBI version of NCBI BlastP web server (Madeira et al. 2024) with default parameters. Full-length sequences of the hits from both searches were retrieved, combined with the corresponding query sequence (LysPM1 or LysPM2), and used to produce multiple sequence alignments with MAFFT (L-INS-i algorithm with default parameters) (Katoh et al. 2017). Multiple sequence alignments were edited in Jalview (Waterhouse et al. 2009) as follows: LysPM1 or LysPM2 was set as reference sequences, the columns with gaps in the reference sequence were hidden, after which the edited multiple sequence alignments were exported in the FASTA format. ESPript was used to visualize the multiple sequence alignments; sequence logos were generated using WebLogo (Crooks et al. 2004; Gouet et al. 1999).

### Determination of lytic activity of LysPM1 and LysPM2

The preparation of the endolysins LysPM1 and LysPM2 was outsourced to an external company (GenScript USA, Inc). The endolysin sequences were back-translated and codon-optimized for expression in *Escherichia coli*, after which chemically synthesized DNA sequences encoding the endolysins with C-terminal 6 × His-tags were cloned into pET-30(a) + using NdeI/HindIII cloning sites, heterologously expressed in *Escherichia coli* BL21 Star (DE3) cells*,* and purified using Ni-NTA IMAC. The purity and protein concentration were determined with the help of SDS-PAGE and Western blot (Figure S1). The purified soluble proteins were used for further experiments.

Turbidity reduction assays were performed to determine the lytic activity of LysPM1 and LysPM2 endolysins using a procedure adapted from previous studies with slight modifications (Zhang et al. 2022). The lytic activity of both endolysins was tested against their phage respective host strains: LysPM1 against *P. mirabilis* Hauser 1885 and LysPM2 against *P. mirabilis* BB2000. Initially, overnight cultures of *P. mirabilis* Hauser 1885 and *P. mirabilis* BB2000 were centrifuged at 3000 × g for 10 min, and the bacterial cell pellets were collected. The collected bacterial cells were then mixed with 1 mL of 0.5 mM EDTA (Invitrogen, Paisley, UK) (final concentration) and incubated for 20 min at room temperature. Subsequently, the EDTA was removed by washing the bacterial pellet three times with 5 mL of PBS buffer. The optical density of the bacterial cells was adjusted to OD_600_ = 1.0 using 50 mM Tris-HCl (pH 8) (Sigma Aldrich). Further, 10 µL (10 µg/mL final concentration) of each endolysin was mixed with 190 µL of the EDTA pre-treated bacterial cells in a 96-well plate. An equivalent volume (10 µL) of lysozyme (10 µg/mL final concentration) was used as a positive control and 10 µL of 50 mM Tris-HCl buffer was used as a negative control. The absorbance at 600 nm was then measured every 10 min for the next 60 min at 37 °C using a BMG Labtech FLUOstar Omega Multi-mode microplate reader.

Following the testing of lytic activity of endolysins, the dose-dependent lytic activity of endolysins was investigated. Both endolysins were mixed with 0.5 mM EDTA pre-treated bacterial cells with different final concentrations of endolysins including 10 µg/mL, 20 µg/mL, 30 µg/mL, and 50 µg/mL and incubated for 60 min in a static incubator at 37 °C. Following the incubation, the absorbance was measured at 600 nm using a BMG Labtech FLUOstar Omega Multi-mode microplate reader.

### Lytic spectrum of LysPM1 and LysPM2 endolysins

The lytic activity of the endolysins LysPM1 and LysPM2 was tested against the strains listed in Table S1. The cell pellets prepared by centrifuging 5 mL of overnight bacterial cultures were treated with 1 mL of 0.5 mM EDTA for 20 min at room temperature. The pellets were washed three times with 5 mL of PBS to remove the EDTA and resuspended in 50 mM Tris–HCl (pH 8). After that, 190 µL of bacterial cell suspensions adjusted to OD_600_ = 1.0 were mixed with 10 µL (10 µg/mL final concentration) of either LysPM1 or LysPM2 in a 96-well microtiter plate and the mixtures were incubated at 37 °C for 60 min. A microplate reader (BMG Labtech FLUOstar Omega) was used to measure the change in OD_600_. The experiments were performed in triplicate.

### pH stability, thermal stability and NaCl stability assays

The pH stability of the endolysins LysPM1 and LysPM2 was determined using a previously described protocol with slight modifications (Cho et al. 2021). Briefly, 50 mM Tris–HCl buffer was adjusted to pH values ranging from 2 to 11 by adding 1 M HCl or 1 M NaOH. Then, endolysins were added to pH adjusted 50 mM Tris–HCl buffer at the final concentration of 10 µg/mL and the reaction mixtures were incubated for 30 min at room temperature. Subsequently, the lytic activity of the endolysins LysPM1 and LysPM2 was assessed on 0.5 mM EDTA pre-treated bacterial cells of *P. mirabilis* Hauser 1885 and *P. mirabilis* BB2000, respectively.

The thermal stability assay of LysPM1 and LysPM2 endolysins was conducted with minor modifications to a previously described method (Heselpoth et al. 2015). A 1 mL aliquot of phage endolysins LysPM1 and LysPM2 at 10 µg/mL final concentration was exposed to a temperature range from 5 to 80 °C for 30 min. Subsequently, the lytic activity of the endolysins LysPM1 and LysPM2 was tested against 0.5 mM EDTA pre-treated bacterial cells of *P. mirabilis* Hauser 1885 and *P. mirabilis* BB2000, respectively.

The effect of NaCl on lytic activity of LysPM1 and LysPM2 endolysins was determined using a previously described method with minor modifications (Etobayeva et al. 2018). Both endolysins at 10 µg/mL final concentration were mixed with 50 mM Tris-HCl containing different final concentrations of NaCl (25 mM, 50 mM, 75 mM, and 100 mM) and incubated for 30 min at room temperature. Following the incubation, the lytic activities of both NaCl-treated endolysins were determined using a turbidity reduction assay against the 0.5 mM EDTA pre-treated bacterial cells of *P. mirabilis* Hauser 1885 and *P. mirabilis* BB2000 for LysPM1 and LysPM2, respectively.

### Testing of phage endolysins using different outer membrane permeabilizers

Turbidity reduction assays were performed to assess the activity of endolysins against the *Proteus* cells using two other membrane-permeabilizing agents, namely citric acid (Sigma Aldrich) and chloroform (Sigma Aldrich) (Liu et al. 2019; Zhang et al. 2022, 2018). Briefly, 5 mL of overnight cultures of *P. mirabilis* BB2000 and *P. mirabilis* Hauser 1885 were centrifuged at 3000 × g for 10 min and pellets collected. The bacterial pellets were resuspended in 1 mL of 0.5 mM citric acid (final concentration) and incubated for 20 min. Subsequently, the cells were pelleted by centrifugation, the supernatant was discarded and the citric acid traces were removed by washing the bacterial pellet three times with 5 mL of PBS buffer.

The endolysins were further tested against chloroform-treated bacterial cells. Overnight cultures (5 mL) of *P. mirabilis* BB2000 and *P. mirabilis* Hauser 1885 were centrifuged at 3000 × g for 10 min and pellets collected. The bacterial pellets were resuspended in 5 mL PBS and chloroform was added to 10% final concentration v/v. The mixtures were then incubated at room temperature for 20 min, followed by a centrifugation at 3000 × g for 10 min. The chloroform-containing supernatants were carefully discarded, and the resulting bacterial pellets were washed with 5 mL of PBS three times.

In addition to testing phage endolysins against chemical permeabilizers, endolysins were also tested against frozen bacterial cells. Overnight cultures (5 mL) of *P. mirabilis* BB2000 and *P. mirabilis* Hauser 1885 were centrifuged at 3000 × g for 10 min, the supernatants were removed, and pellets collected. The obtained bacterial pellets were washed with 5 mL PBS three times and stored at − 80 °C for 24 h.

The optical density of the EDTA-treated,citric-acid-treated, chloroform-treated, and frozen bacterial cells was adjusted to OD_600_ = 1.0 to 1.2 using 50 mM Tris-HCl (pH 8). Further, 10 µL (10 µg/mL final concentration) of each endolysin was mixed with 190 µL of the previously optical-density-adjusted bacterial cells in a 96-well plate. The reaction mixtures were incubated at 37 °C for 60 min in a static incubator and absorbance was then measured at 600 nm using a BMG Labtech FLUOstar Omega Multi-mode microplate reader. The results were expressed as relative lytic activity (%) using the formula adopted from (Zhang et al. 2021):

$$\mathrm{Relative}\;\mathrm{lytic}\;\mathrm{activity}\;(\%)\hspace{0.17em}=\hspace{0.17em}(\Delta{\mathrm{OD}}_{600\;\mathrm{buffer}}-\Delta{\mathrm{OD}}_{600\;\mathrm{endolysin}})/{\mathrm{OD}}_{600\;\mathrm{initial}}\hspace{0.17em}\times\hspace{0.17em}100$$

### Determination of the effect of metal ions on the lytic activity of endolysins

The effect of metal ions on the lytic activity of endolysins was determined as previously reported with minor modifications (Ding et al. 2020). To remove the metal ions attached to endolysins, 5 mM EDTA (final concentration) was mixed with 500 µL of endolysin (200 µg/mL) and incubated for 30 min at room temperature. Next, 10 µL (10 µg/mL final concentration) of the EDTA-treated endolysins LysPM1 and LysPM2 were mixed with 190 µL of 0.5 mM EDTA pre-treated bacterial cells of *P. mirabilis* Hauser 1885 and *P. mirabilis* BB2000, respectively. The cell suspensions were supplemented with metal ions at 1 mM final concentration (CaCl_2_, MnCl_2_, ZnSO_4_, MgCl_2_). Then, the mixtures were incubated for 60 min at 37 °C in static condition, and absorbance was measured at 600 nm using a BMG Labtech FLUOstar Omega multi-mode microplate reader.

## Results

### In silico analysis

**Fig. 1:**
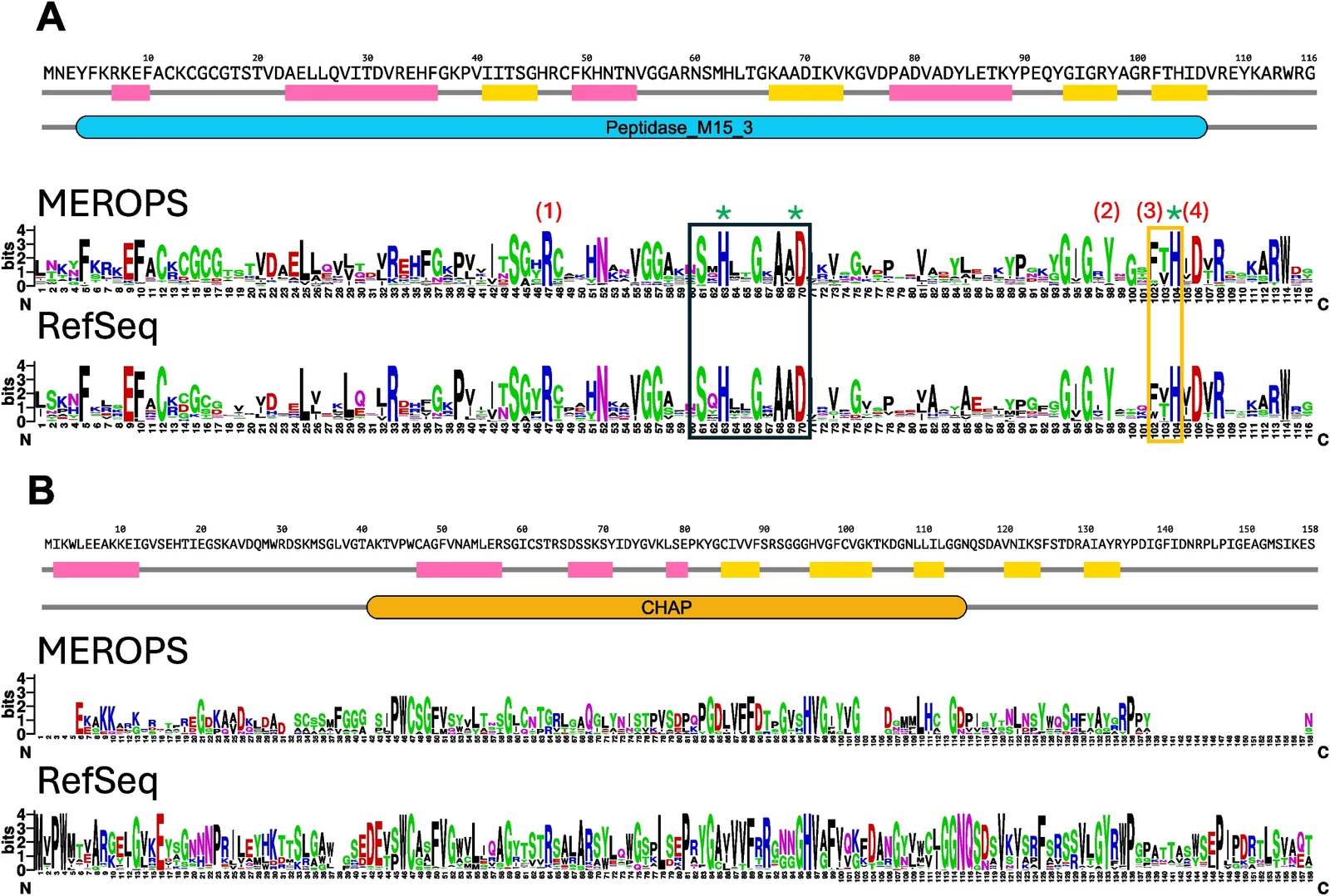
Sequence analysis of endolysins LysPM1 (**A**) and LysPM2 (**B**). Protein domain architecture and secondary structure are shown above sequence logos. Secondary structure prediction was performed in PSIPRED; helices are coloured in pink, strands in yellow. Multiple sequence alignments with hits from MEROPS and viral RefSeq databases were generated by MAFFT and edited in Jalview. The logos were produced using WebLogo. The height of the letters represents the conservation of the amino acid at that position. The red numbers indicate specific functionally relevant residues further discussed in the main text. The black box highlights the conserved motif SxH*xxGxAxD* found in zinc hydrolases, with asterisks indicating amino acid ligands of the zinc atom; the orange box highlights the presence of a catalytic motif (W/F)xH

The endolysin LysPM1 from *Proteus* phage Homer is a 116 aa-long protein with a deduced molecular weight of approximately 13.1 kDa, containing a single peptidase_M15_3 domain (IPR013230; Peptidase M15A, C-terminal). The LysPM2 endolysin was identified in *Proteus* phage PM-CJR as a 158 aa-long protein (GenBank: QYC51792) with approximate molecular weight of 17.11 kDa. InterProScan analysis predicted that LysPM2 endolysin contained a single CHAP domain (IPR007921). No other functional domains were detected in either of the endolysins LysPM1 or LysPM2 (Fig. 1A and B).

**Fig. 2:**
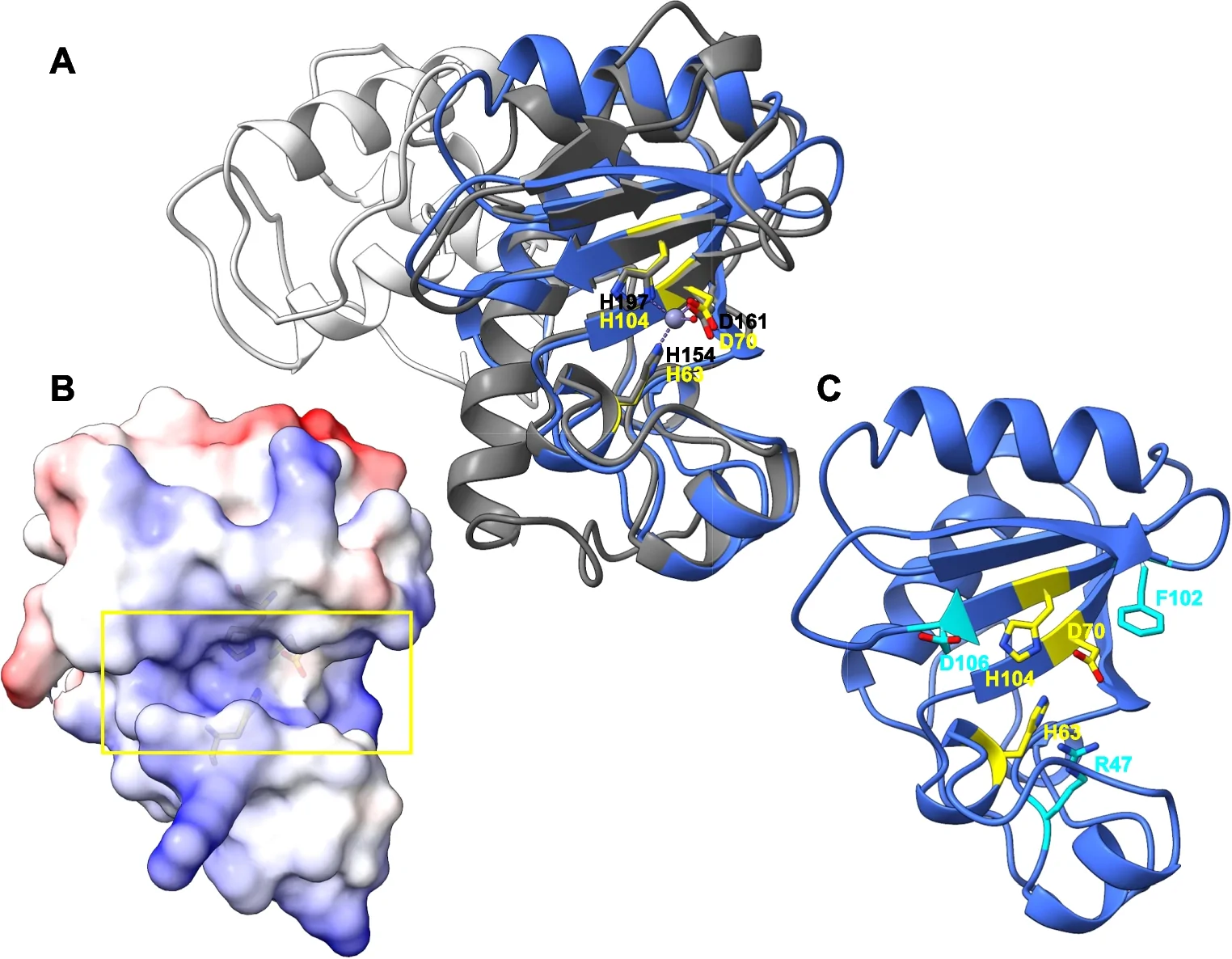
Structural analysis of LysPM1. **A** Superposition of LysPM1 model and Zn^2+^-D-Ala-D-Ala carboxypeptidase from *Streptomyces albus* G (Charlier et al. 2011), (PDB ID: 1LBU). LysPM1 model is coloured in blue, 1LBU is coloured in dark grey (C-terminal domain) and in light grey (N-terminal domain). Residues responsible for Zn^2+^ binding in 1LBU (H154, D161, H197) are shown; matching residues in LysPM1 are coloured in yellow. **B** The electrostatic surface representation of LysPM1; red represents negative charge, and blue represents positive charge. The active site is framed by a yellow rectangle. **C** Close-up view of the active site of LysPM1. Residues predicted to be involved in Zn^2+^-binding (H63, D70, H104) are coloured in yellow; potentially catalytic residues (R47, D106, F102) are coloured in cyan

**Fig. 3:**
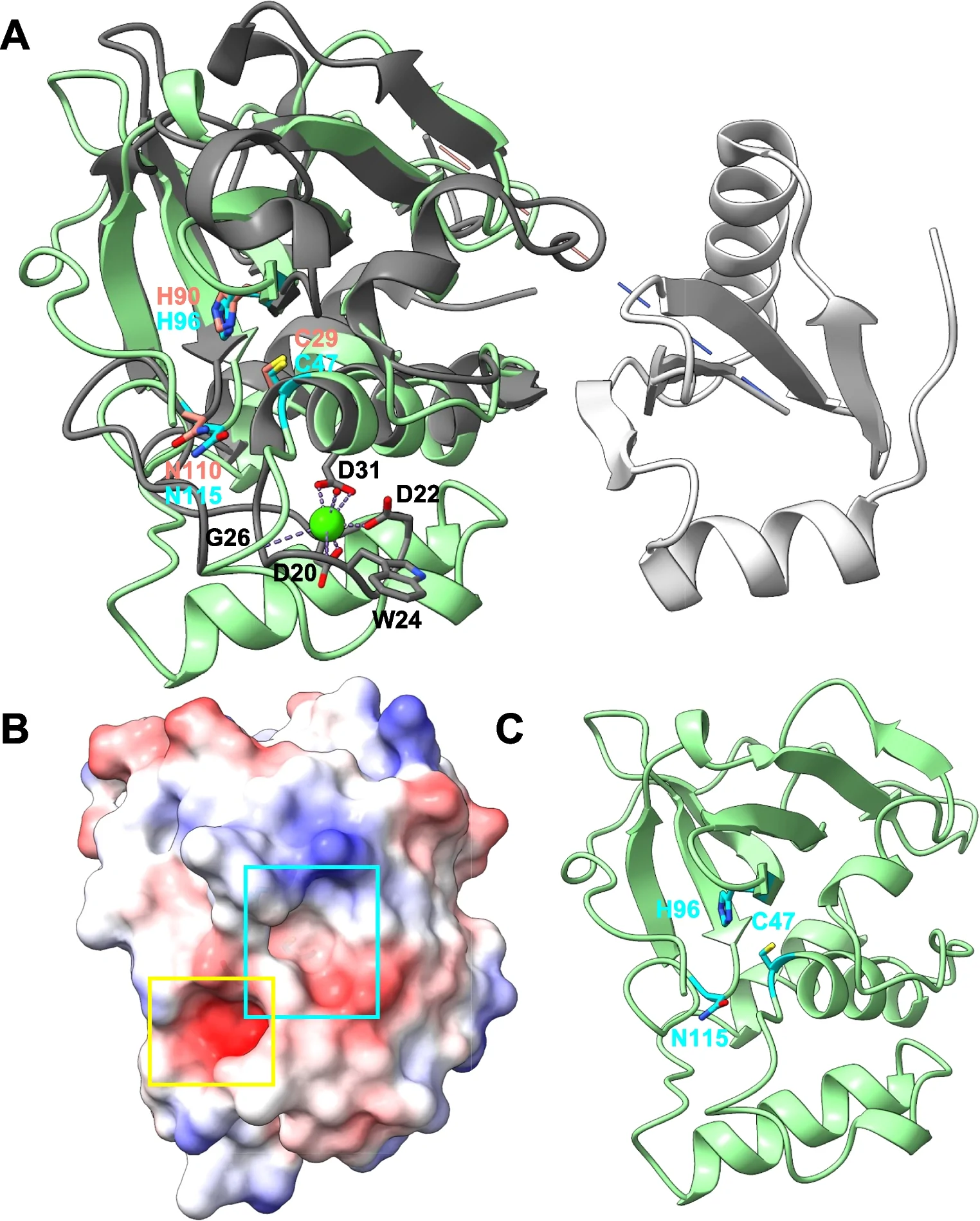
Structural analysis of LysPM2. **A** Superposition of LysPM2 model and endolysin LysIME-EF1 from the *Enterococcus faecalis* phage IME-EF1 (Zhou et al. 2020). LysPM2 model is coloured in green, 6IST is coloured in dark grey (N-terminal domain) and light grey (C-terminal domain). Residues responsible for Ca^2+^ binding in 6IST (D20, D22, W24, G26, D31) are shown. Catalytic residues (C29, H90, N110) in 6IST are coloured in salmon, matching residues in LysPM2 are coloured in cyan. **B** The electrostatic surface representation of LysPM2. Red represents negative charge, and blue represents positive charge. The active site is indicated by a cyan rectangle, the potential Ca^2+^-binding site is indicated by a yellow rectangle. **C** Close-up view of the active site of LysPM2, the residues of the predicted catalytic triad (C47, H96, N115) are labelled in cyan

Multiple sequence alignments of LysPM1 endolysin and related enzymes identified through a BLASTp search against viral RefSeq and MEROPS-MP databases were performed to identify conserved residues; this procedure was repeated to produce multiple sequence alignments of LysPM2 (Figure S2). Conservation of amino acids at each position was represented as sequence logos (Fig. 1). AlphaFold 3 models of the endolysins were generated and visualized with the help of ChimeraX (Figs. 2 and 3). DALI webserver was used to identify the best structurally matching experimentally determined protein structures in the PDB. The structural comparisons conducted in ChimeraX in combination with the amino acid conservation analysis allowed us to identify residues in LysPM1 and LysPM2 matching those previously determined to be catalytic and/or involved in binding of metal ions.

The top structural hit for LysPM1 was to 1LBU (Z-score 14.2), Zn^2+^-D-Ala-D-Ala carboxypeptidase from *Streptomyces albus* G and the type enzyme of the M15A subfamily (Charlier et al. 2011), which was thus selected for structural comparisons. Key active site residues and a probable catalytic mechanism for 1LBU were previously described (Charlier et al. 2025, 2011; Mechanism and Catalytic Site Atlas n.d.). We identified the Zn^2+^-binding triad (H63, D70, H104) in LysPM1 (Fig. 1A, green asterisks); H63 and D70 are found in the previously described motif SxH*xxGxAxD* (aa* being ligands of the zinc atom) typical of this class of zinc hydrolases (Fig. 1A, black box). Two conserved residues R47 and Y98 (Fig. 1A, (1) and (2)) are also of interest. The residue R47 corresponds to R138 in 1LBU, which is predicted to be involved in the initial electrostatic interactions with the substrate terminal carboxylate, while Y98 is positioned approximately in the same region where Y189 is found in 1LBU (Charlier et al. 2011).

In the case of LysPM2, as several top hits had comparable Z-scores (between 11 and 12), 6IST (crystal structure of a wild-type endolysin LysIME-EF1 from the *Enterococcus faecalis* phage IME-EF1) was chosen as the most biologically relevant (Zhou et al. 2020). The catalytic triad of LysPM2 was predicted to be formed by C47, H96, and N115, corresponding to catalytic residues C29, H90, and N110 forming an amidohydrolase active site in 6IST. The endolysin LysIME-EF1 also contains a single Ca^2+^ ion, coordinated by five conserved residues (D20, D22, W24, G26, D31) and one water molecule. Although our tests suggest that the lytic activity of LysPM2 relies on the presence of Ca^2+^ (or Mn^2+^) cations (see below), there is a substantial structural difference between the region responsible for binding of Ca^2+^ in 6IST and the corresponding region of the LysPM2 model (Fig. 3A), which makes it difficult to reliably determine the residues responsible for Ca^2+^ coordination in LysPM2. A cavity rich in negatively charged residues is present in LysPM2 (Fig. 3B), which potentially could be the Ca^2+^-binding site, but experimental verification of this hypothesis is required.

### Determination of lytic activity of LysPM1 and LysPM2

**Fig. 4:**
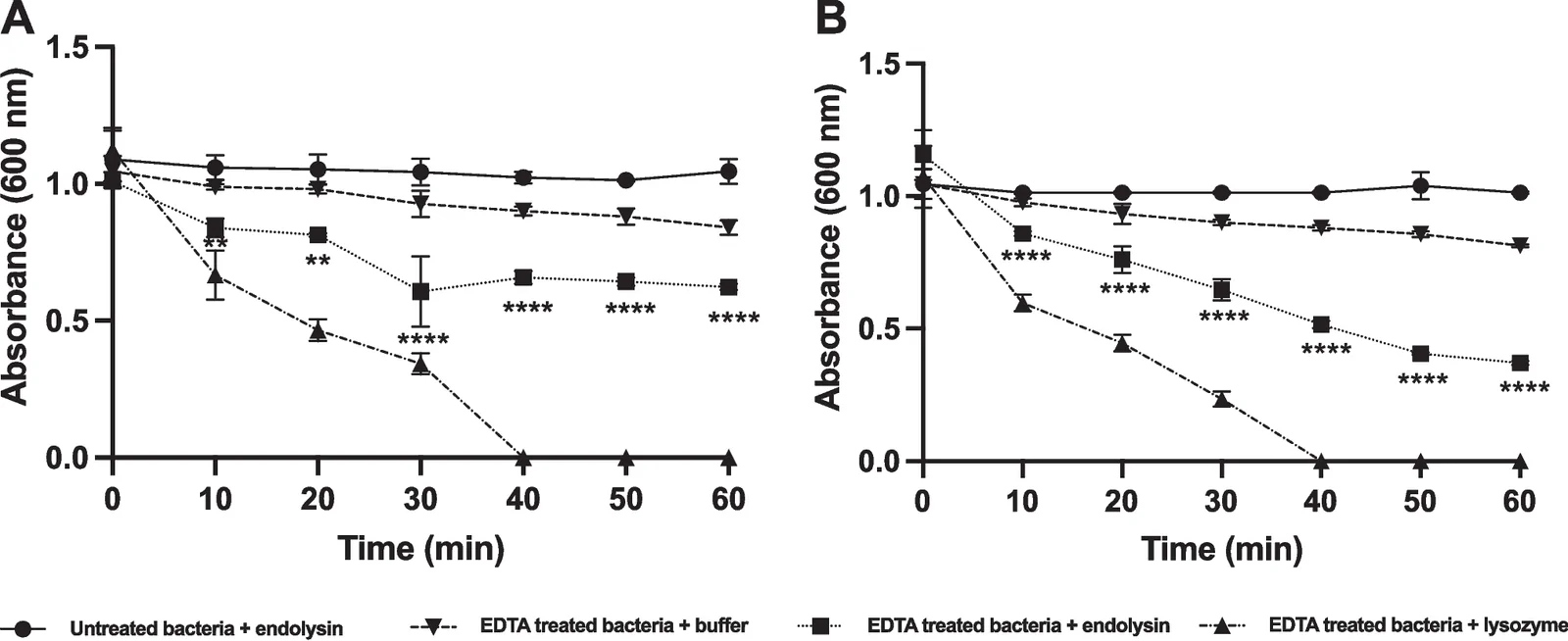
Lytic activity of endolysins LysPM1 (**A**) and LysPM2 (**B**) against *P. mirabilis* Hauser 1885 and *P. mirabilis* BB2000, respectively. Final concentration of endolysins and lysozyme in the reactions–10 µg/mL. Mean values of three biological replicates are plotted; error bars represent ± standard deviation. Asterisks indicate significant differences between the relevant exposure times compared to baseline (time = 0), *(*p* < 0.05), **(*p* < 0.01), ***(*p* < 0.001), and ****(*p* < 0.0001) using one-way ANOVA and Dunnett’s post-test analysis

The recombinant endolysins LysPM1 and LysPM2 were supplied by GenScript alongside the SDS-PAGE and Western blot results, confirming that the enzymes were of expected size and acceptable purity for further biochemical characterization (Figure S1). Both endolysins were supplied in the following buffer: 50 mM Tris-HCl, 500 mM NaCl, 10% Glycerol, pH 8.0. LysPM1 concentration was 0.99 mg/mL; LysPM2 concentration was 1.45 mg/mL. The endolysins were supplied on dry ice and stored at −80ºC. Bacteriolytic activity of the endolysins received was assessed using the turbidity reduction assays against cell suspensions of *P. mirabilis* Hauser 1885 and *P. mirabilis* BB2000, which are the hosts of the phages Homer (LysPM1 source) and PM-CJR (LysPM2 source), respectively. The addition of LysPM1 (Fig. 4A) to untreated bacterial cells of *P. mirabilis* Hauser 1885 showed no changes in absorbance over the incubation period. However, the optical density of the EDTA pre-treated *P. mirabilis* Hauser 1885 cell suspension declined from 1.0 to 0.62 within 60 min. Similarly, when LysPM2 (Fig. 4B) was tested against EDTA-treated and untreated *P. mirabilis* strain BB2000, no lytic activity could be detected against the untreated *P. mirabilis* BB2000 culture, whereas LysPM2 addition to EDTA pre-treated bacterial cells caused a steady decline in turbidity over time, decreasing from 1.0 to 0.37 within 60 min. For both endolysins, lysozyme was used as a positive control, reducing the turbidity of both bacterial suspensions to 0 within 40 min. The EDTA treatment alone of both *P. mirabilis* Hauser 1885 and *P. mirabilis* BB2000 cell suspensions resulted in a smaller change in optical density (from 1.10 to around 0.80) compared to EDTA and endolysin-treated samples.

Following the initial confirmation of lytic activity, different final concentrations (0, 10, 20, 30, and 50 µg/mL) of both endolysins were added to EDTA pre-treated cells and incubated at 37 °C for 60 min. Higher concentrations of each endolysin resulted in more effective bacterial lysis, indicating a dose-dependent lytic activity (Figure S3).

### Lytic spectrum of LysPM1 and LysPM2 endolysin

Turbidity reduction assays were conducted to determine the lytic spectra of LysPM1 and LysPM2 endolysins against a panel of EDTA (0.5 mM) pre-treated bacterial strains and compare them to the host ranges of their respective phages. As could be seen from Table 1, LysPM1 was active against seven *P. mirabilis* strains and one strain of *P. vulgaris*, while Homer could only infect two strains of *P. mirabilis*. Host range of PM-CJR and the lytic spectrum of its endolysin LysPM2 were found to be identical, constituting nine *P. mirabilis* strains, one strain of *P. vulgaris*, and one strain of *Proteus penneri*.

**Table 1.**
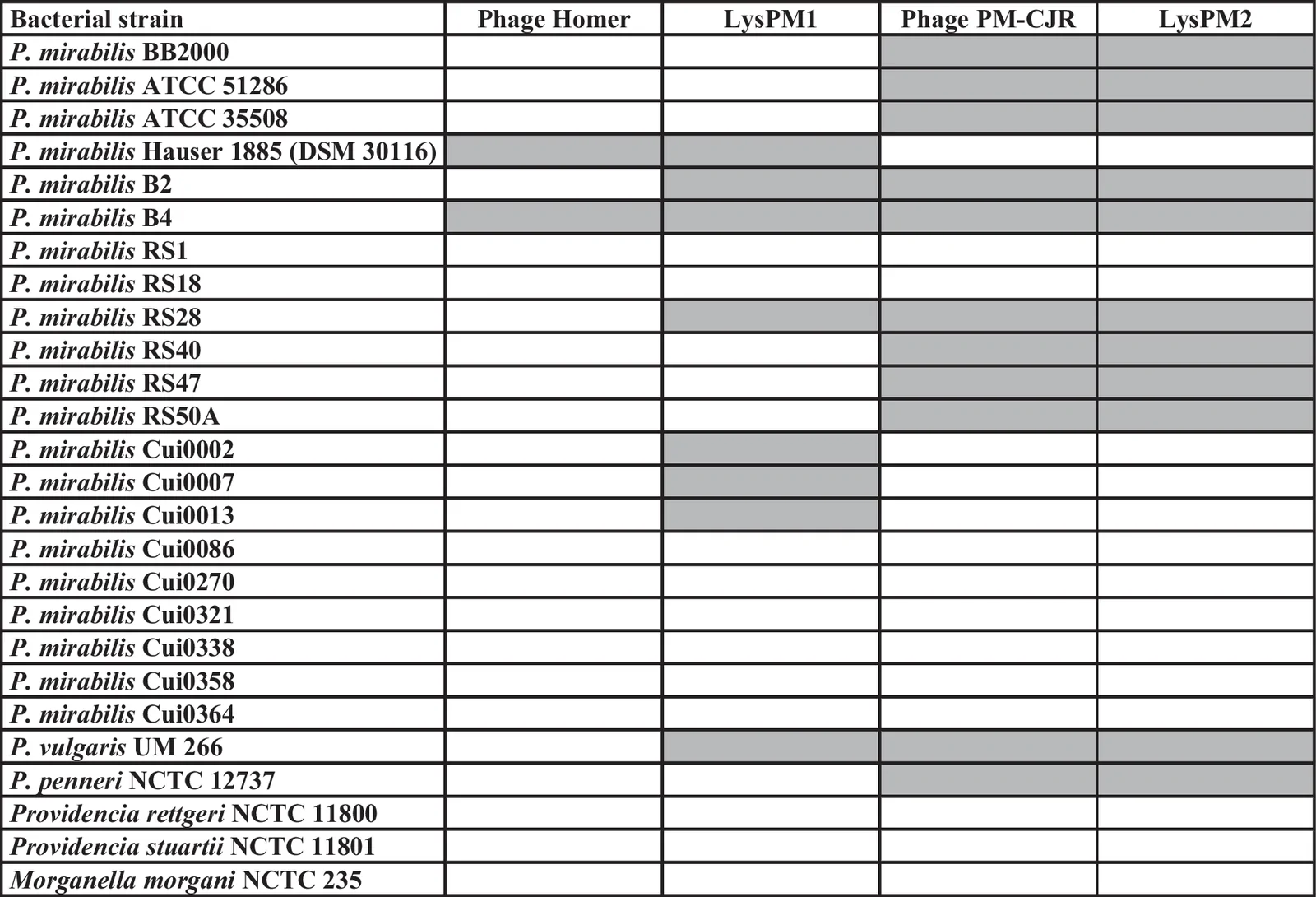
Lytic spectra of LysPM1 and LysPM2 endolysins and the phages they originated from. Shaded cells indicate that the phage or endolysin demonstrates lytic activity against the respective bacterial strain

### pH, thermal and NaCl stability assays

The pH stability of LysPM1 and LysPM2 was assessed next by comparing the lytic activity of the endolysins against EDTA pre-treated cells at pH 2–11. Optimum pH was determined to be between 7 and 8, with the lytic activity of both endolysins negatively affected at acidic and basic pH levels (Figure S4A). LysPM1 demonstrated somewhat greater stability across the entire pH range tested compared to LysPM2, and both endolysins completely lost their lytic activity at pH 2.

Thermal stability of LysPM1 and LysPM2 was evaluated by incubating the endolysins across a range of temperatures from 5 to 80 °C, followed by the assessment of their lytic activity against EDTA pre-treated bacterial cells. Endolysins remained stable when tested at temperatures up to 40 °C, above which the enzymes started losing their lytic activity, with complete inactivation at 70 °C (Figure S4B).

Finally, each of the enzymes was tested over a range of NaCl concentrations. In Figure S4C, there was no significant difference in the lytic activity of LysPM1 and LysPM2 endolysins in the presence of up to 100 mM NaCl.

### Testing of phage endolysins using different outer membrane permeabilizers

**Fig. 5:**
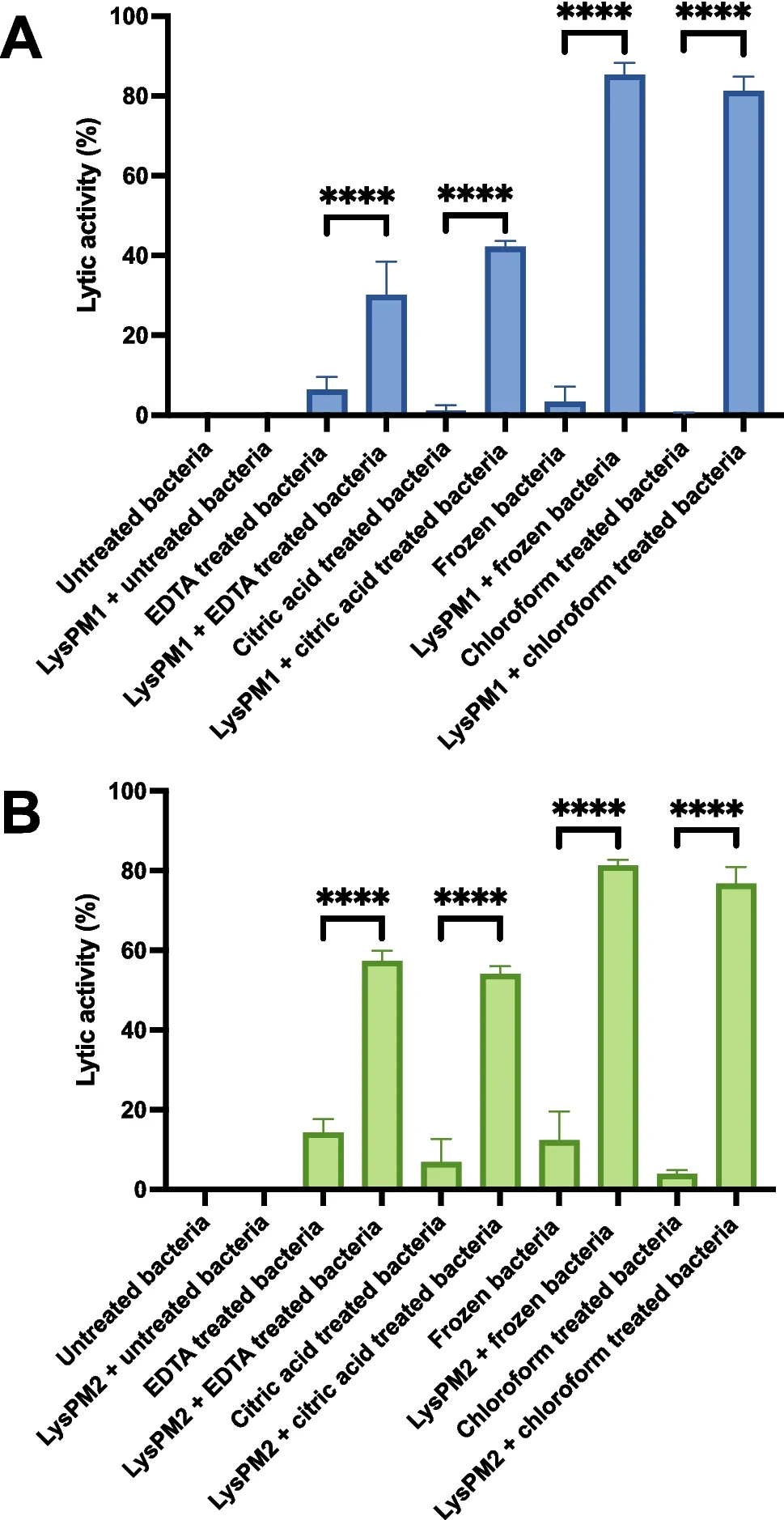
The effect of several outer membrane permeabilization techniques on the lytic activity of phage endolysins LysPM1 (**A**) and LysPM2 (**B**) assayed against *P. mirabilis* Hauser 1885 and *P. mirabilis* BB2000, respectively. Bacterial pellets were either frozen at –80 °C or pre-treated with EDTA (0.5 mM), citric acid (0.5 mM), and chloroform (10% v/v) before treatment with 10 µg/mL endolysin (final concentration). Mean values of three biological replicates are plotted; error bars represent ± standard deviation. Asterisks indicate significant differences between the control (pre-treated bacteria only) and the respective test (pre-treated bacteria + endolysin) groups: *(*p* < 0.05), **(*p* < 0.01), ***(*p* < 0.001), and ****(*p* < 0.0001). Statistical testing was performed using one-way ANOVA and Tukey’s multiple comparisons test (*n* = 3)

The effect of several outer membrane permeabilization techniques on the lytic activity of phage endolysins was determined next. Bacterial pellets were either frozen at –80 °C or pre-treated with EDTA (0.5 mM), citric acid (0.5 mM), and chloroform (10% v/v). The lytic activity of LysPM1 and LysPM2 endolysins was then assessed using the turbidity reduction assay as described in the Materials and Methods. In the testing of LysPM1 (Fig. 5A), the highest lytic activity was observed against frozen bacterial pellets (85%) and chloroform-treated cells (81%), while the pretreatment with EDTA and citric acid resulted in 30% and 42% lytic activity, respectively. In the case of LysPM2 (Fig. 5B), the highest reduction in turbidity was again observed in the test with frozen bacterial cells (around 81%), followed by the chloroform-treated cells (around 76%). The optical density of EDTA-treated cells in combination with endolysin decreased approximately 57%, while the pretreatment with citric acid had the least substantial effect, resulting in turbidity reduction of around 54%.

### Metal ion requirements of the endolysins LysPM1 and LysPM2

**Fig. 6:**
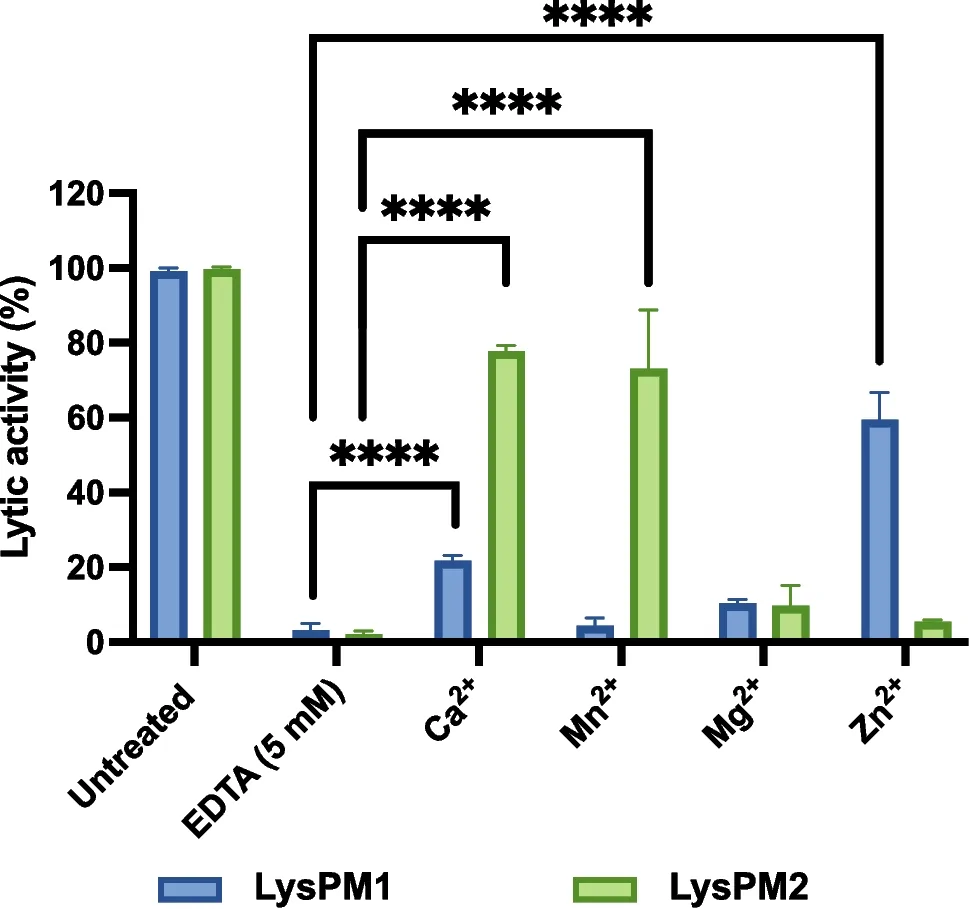
The effects of different concentrations of divalent cations on the lytic activity of endolysins LysPM1 and LysPM2. Mean values of three biological replicates are plotted; error bars represent ± standard deviation. Asterisks indicate significant differences between the control (EDTA treated endolysin) and the respective test (EDTA treated endolysin supplied with a specific divalent cation) groups: *(*p* < 0.05), **(*p* < 0.01), ***(*p* < 0.001), and ****(*p* < 0.0001). Statistical testing was performed using one-way ANOVA and Dunnett's multiple comparisons test (*n* = 3)

The effect of metal ions on the lytic activity of LysPM1 and LysPM2 endolysins was evaluated. As illustrated in Fig. 6, almost complete loss of lytic activity was observed for both endolysins in the presence of EDTA (5 mM). However, the addition of divalent metal salts to a final concentration of 1 mM resulted in a partial recovery of enzymatic activity even in the presence of 0.25 mM residual EDTA. Specifically, the lytic activity of EDTA-treated LysPM1 endolysin was partially restored by Zn2⁺, with approximately 59% recovery, and the addition of Ca2⁺ source resulted in a recovery of approximately 21% of lytic activity, whereas the addition of Mn2⁺ and Mg2⁺ salts had a negligible effect. In contrast, the lytic activity of the EDTA-treated LysPM2 endolysin was most effectively restored by Ca2⁺, with a recovery of nearly 77%. Mn2⁺ addition resulted in around 73% recovery, while Mg2⁺ and Zn2⁺ did not lead to any substantial recovery of the lytic activity.

## Discussion

Endolysins from phages infecting Gram-negative hosts are usually small and globular, typically containing only a single enzymatically active domain but no cell-binding domains, unlike the multidomain endolysins of Gram-positive phages (Schmelcher et al. 2012); *Proteus* phage endolysins LysPM1 and LysPM2 follow this pattern.

LysPM1 was classified as a metallopeptidase of the subfamily M15A. To the best of our knowledge, only two phage endolysins belonging to the M15A subfamily have been experimentally verified to date: the peptidase M15A from *Burkholderia pseudomallei* phage ST79 (Khakhum et al. 2016a, 2016b) and the lytic protein Spp64 from the *Shewanella* bacteriophage Spp001 (Zhang et al. 2018). In both cases, the authors’ propose that Phe or Trp in the motif (W/F)xH could be a catalytic residue. LysPM1 also contains a conserved F102 residue. As it is positionally corresponding to H195 in 1LBU, we believe that it is unlikely that F102 is directly involved in catalysis, given the role of H195 as a general base, and speculate that it might be involved in hydrophobic interactions with the substrate instead, analogous to the role of W114 in EndoT5 (Mikoulinskaia et al. 2023). The presence of an acidic residue, aspartic or glutamic acid, is required in the active site of zinc peptidases to activate the zinc-bound water molecule, allowing the catalytic cleavage of the target peptide to take place (Charlier et al. 2011). The only acidic residue in the vicinity of the active site of LysPM1 is D106, suggesting that it could be directly or indirectly involved in proton transfer, especially given its conservation in proteins similar to LysPM1.

LysPM2 contains a domain belonging to a superfamily of cysteine, histidine-dependent amidohydrolases/peptidases (CHAP). CHAP domains are typically found in enzymes involved in cell-wall hydrolysis and have been unified under the peptidase families C40 and C51 in the MEROPS database (Faheem et al. 2016). CHAP domains contain an invariant cysteine and histidine residue in their active site for substrate cleavage and are classified based on the molecular mechanism by which the peptidoglycan cleavage occurs rather than the specific bond cleaved (Broendum et al. 2018; Schmelcher et al. 2012); some CHAP have amidase activity, while others act as endopeptidases. CHAP domains are commonly found in endolysins of Gram-positive phages (Liu et al. 2023; Oliveira et al. 2013), but endolysins of some Gram-negative phages contain them as well (Wang et al. 2022), albeit rarely.

Endolysins typically exhibit limited activity when applied against Gram-negative species, requiring the use of external outer membrane permeabilizing agents to disrupt this outer layer and enable the endolysin to access and degrade the peptidoglycan layer beneath it effectively (Briers et al. 2011). Although a few endolysins reported in the literature have an intrinsic antibacterial activity against Gram-negative bacteria (Sisson et al. 2024), the bioinformatics analysis demonstrated that the currently known features of such enzymes (amphipathic α-helices, polycationic tails, signal-anchor-release (SAR) domain) were present in neither LysPM1 nor LysPM2.

Both endolysins were inactive against untreated bacteria, but exhibited a dose-dependent lytic activity when tested against EDTA pre-treated *Proteus* cells, but not other Gram-negative bacteria, even closely related *Morganella* and *Providencia.* LysPM1 demonstrated a narrower lytic spectrum, lysing eight *Proteus* strains, while its phage Homer only infected two *Proteus* strains. Upon testing of the lytic spectrum of LysPM2, it was observed that LysPM2 lysed the same *Proteus* strains that its phage PM-CJR infected, 11 strains in total. Typically, endolysins possess a broader spectrum of lytic activity compared to their respective phages (Deng et al. 2023; Jiang et al. 2021; Lu et al. 2023). This can be explained by the relatively higher conservation of the peptidoglycan structure compared to the diversity of mechanisms that prevent phage infection, such as the presence of highly diverse phage defence systems and variability in bacterial cell surface receptors, which restrict phage host range. A plausible explanation is that strains that could be lysed by LysPM2 but could not be infected by its host phage, PM-CJR, were not amongst the strains tested. Finally, three *Proteus* strains (*P. mirabilis* B2*, P. mirabilis* B4*,* and *P. vulgaris* UM 266) were susceptible to lysis by both LysPM1 and LysPM2, suggesting similar composition of their cell walls. The strain-specific differences in the host range of LysPM1 and LysPM2 may reflect variation in peptidoglycan composition or modification between strains. For example, O-acetylation has previously been reported in the peptidoglycan of *Proteus mirabilis* (Dupont and Clarke 1991) and may influence susceptibility to muralytic enzymes. Differences in the extent or distribution of such modifications could therefore contribute to the lytic patterns observed in this study.

Although no other *Proteus* phage-derived endolysins have been experimentally tested, several non-*Proteus* endolysins with various levels of activity against *Proteus* spp. have been reported in literature, such as Lys46_30_ isolated from a prophage in *Bacillus subtilis* strain DDBCC46 and *Escherichia coli* T5 phage endolysin (Mikoulinskaia et al. 2009; Sarjoughian et al. 2020). Nevertheless, direct comparisons of the effectiveness of LysPM1 and LysPM2 are challenging due to the lack of standardization in endolysin assays. Both LysPM1 and LysPM2 endolysins were active at a concentration of 10 µg/mL, demonstrated moderate thermal and pH stability and were not noticeably affected by adjusting the ionic strength of the reaction buffer, comparable to the majority of the endolysins experimentally characterized to date.

Several outer membrane permeabilizers were utilized to assess their impact on the lytic activity of LysPM1 and LysPM2 endolysins. Both enzymes demonstrated maximum lytic strength against chloroform-treated and frozen bacteria, while EDTA and citric acid treated cells were less affected by endolysins. This can be explained by more efficient disruption of outer membranes by organic solvent and damage from freeze-thawing of cells compared to milder organic acid treatment. Alternatively, it is possible that, despite washing the bacterial pellet three times to remove EDTA and citric acid, some traces of these metal-ion chelators might have remained, affecting the lytic activity of LysPM1 endolysin, predicted to be a M15A metalloenzyme with a Zn2⁺ cofactor in the active site. In the case of LysPM2, more prominent reduction of turbidity of suspensions EDTA and citric acid pre-treated cells was observed compared to LysPM1. CHAP enzymes do not rely on divalent ions for their catalytic activity, but metal ions, such as calcium or magnesium, might be required for the structural integrity of the enzyme or to facilitate substrate binding.

To test the hypothesis regarding the metal-dependent lytic activity of LysPM1 and LysPM2 endolysins, both enzymes were incubated with 5 mM EDTA, after which metal ions were added to assess the restoration of lytic activity. Both enzymes were almost completely inactivated by EDTA. The subsequent addition of either Zn2⁺ or Ca2⁺ (but not Mg2⁺ or Mn2⁺) partially restored LysPM1 activity. In previously published reports, EDTA completely inactivated M15C metallopeptidases from Gram-negative *Escherichia* (EndoT5) and *Salmonella* (LysSTG2), but the addition of divalent Ca2⁺ (but not Zn2⁺) partially restored their activity (Mikoulinskaia et al. 2009; Zhang et al. 2021). While we demonstrate that Zn^2+^ addition helps to restore LysPM1 activity, in a previous study of another peptidase M15A from phage ST79 of Gram-negative *Burkholderia pseudomallei* it was reported that endolysin activity was reduced by the presence of Zn^2+^. Given that the addition of Ca^2+^ resulted in a non-negligible restoration of LysPM1 activity, it might indicate that LysPM1 could potentially be Ca^2+^-regulated, similar to the endolysin of bacteriophage T5 (EndoT5), which is a Zn^2+^-dependent and Ca^2+^-activated L-alanyl-D-glutamate peptidase M15C (Prokhorov et al. 2024).

In the case of LysPM2, the addition of Ca2⁺ and Mn2⁺ ions significantly restored its activity lost after EDTA treatment. While Mn^2+^ is unlikely to be the natural cofactor, Mn^2+^ can substitute for Ca^2+^ in certain situations due to similarities in both valence and atomic radius. The addition of Mg2⁺ or Zn2⁺ to the EDTA-treated endolysin LysPM2 did not restore its activity. LysPM2 is a CHAP domain-containing endolysin; some experimentally characterized endolysins with CHAP domains include CHAP_k_ and PlyGRCS (Fenton et al. 2011; Linden et al. 2014) from Gram-positive *Staphylococcus aureus* phages and Lysqdvp001 from a phage infecting Gram-negative *Vibrio parahaemolyticus* (Wang et al. 2016). Although the metal dependence of Lysqdvp001 was not established, endolysins CHAP_k_ and PlyGRCS were both reported to be dependent on Ca2⁺ cations for their lytic activity. Interestingly, the presence of a Zn^2+^ cation was also detected in the crystal structure of CHAP_k_, although it appears to be loosely bound and presumably having a regulatory function (Sanz-Gaitero et al. 2014). Finally, it should be noted the addition of Mg^2+^ did not substantially restore the lytic activity of either endolysin. One possible explanation is that Mg2⁺ partially stabilized the bacterial outer membrane (Clifton et al. 2015), thereby reducing endolysin penetration following EDTA pretreatment.

## Conclusion

We described and characterized two *Proteus* phage endolysins, which demonstrated lytic activity against an array of *Proteus* species in combination with outer membrane permeabilizing agents. LysPM1 is only the third experimentally characterized Peptidase M15A from a Gram-negative phage, while LysPM2 is not only a rare example of a CHAP-containing endolysin from a Gram-negative phage but, to the best of our knowledge, is the first example of an experimentally characterized single-domain Gram-negative endolysin containing a CHAP domain. The findings presented in this study improve our understanding of Gram-negative endolysins and may contribute to the development of new approaches to fight infections caused by *P. mirabilis*. In future, these endolysins could be further characterized, including having their tertiary structure verified using X-ray crystallography and/or cryo-EM, and investigated against *P. mirabilis* biofilms alone and/or in combination with other antibacterial agents such as antibiotics or cold plasma-activated water, which was shown to synergize with phages when used against *Proteus* biofilms (Shambharkar et al. 2024). In addition, these endolysins can be chemically modified and/or genetically engineered to enhance their activity and stability, as well as to broaden their spectrum against Gram-negative bacteria. For example, fusing outer membrane permeabilizing peptides to endolysins can improve their ability to penetrate the outer membrane of Gram-negative bacteria without the need for chemical permeabilisers, offering a more practical approach for their application.

## Supplementary Information

Below is the link to the electronic supplementary material.

ESM 1 (PDF 779 KB)

## Data Availability

All data generated or analyzed during this study are presented in the manuscript text and/or Supporting Information.
