# Supplementary material for: First experimentally characterised Proteus phage endolysins have distinct metal cofactor dependencies
Source: Appl Microbiol Biotechnol. 2026 Jun 6;110:243. doi: 10.1007/s00253-026-13891-1 (PMC13461873; doi:10.1007/s00253-026-13891-1)
Supplement: Supplementary file 1 [file 253_2026_13891_MOESM1_ESM.pdf]

First experimentally characterised *Proteus* phage endolysins have distinct metal cofactor dependencies

Applied Microbiology and Biotechnology

Akash Shambharkar[1], Thomas P. Thompson[1], Laura A. McClenaghan[1], Katie J. J. Brown[1], Stephen A. Kelly[1,2], Brendan F. Gilmore[1,2], Timofey Skvortsov[1*]

[1] School of Pharmacy, Queen's University Belfast, Medical Biology Centre, 97 Lisburn Road, Belfast, BT9 7BL, UK

[2] School of Medicine and Centre for Interventions in Infection, Inflammation, and Immunity (4i), University of Limerick, Limerick, Ireland.

ashambharkar01@qub.ac.uk (0009-0008-3452-7309)

T.Thompson@qub.ac.uk (0000-0001-8125-2303)

l.mcclenaghan@qub.ac.uk (0009-0008-5041-438X)

kbrown52@qub.ac.uk (0009-0007-0054-7603)

Stephen.Kelly@qub.ac.uk (0000-0002-8907-9661)

b.gilmore@qub.ac.uk (0000-0001-7675-9062)

t.skvortsov@qub.ac.uk (0000-0002-4048-6725)

* corresponding author

Captions

Supplementary tables

Table S1. Bacterial strains used for host range analysis.

Table S2. Protein sequences of endolysins LysPM1 and LysPM2.

Supplementary figures

Figure S1. The SDS-PAGE and Western blot (with mouse anti-His-tag antibodies) results provided by GenScript. (A) LysPM1 and (B) LysPM2 endolysin. In SDS-PAGE: M_1 – protein marker, BSA – bovine serum albumin (2 µg), R – purified endolysin sample (reducing conditions). In Western blot: M_2 – protein marker, R – purified endolysin sample (reducing conditions).

Figure S2. Multiple sequence alignments of endolysins LysPM1 (A) and LysPM2 (B) with BlastP hits from MEROPS and RefSeq databases produced by MAFFT, edited in Jalview, and visualised by ESPript.

Figure S3. Dose dependent lytic activity of endolysins LysPM1 (A) and LysPM2 (B) against *P. mirabilis* Hauser 1885 and *P. mirabilis* BB2000, respectively. Mean values of three biological replicates are plotted; error bars represent ± standard deviation. Asterisks indicate significant differences between the relevant treatment groups compared to baseline (0 µg/mL) and each treatment group, * ($p < 0.05$), **($p < 0.01$), ***, ($p < 0.001$), and **** ($p < 0.0001$) using one-way ANOVA and Tukey's multiple comparisons test analysis ($n = 3$).

Figure S4. Endolysin stability assays. (A) The pH stability testing of endolysins LysPM1 and LysPM2 against the 0.5 mM EDTA pre-treated cell suspensions of *P. mirabilis* Hauser 1885 and *P. mirabilis* BB2000, respectively. The mean relative activity values are plotted; the error bars represent ± standard deviation from three different biological and three technical replicates. (B) The temperature stability assay of endolysins LysPM1 and LysPM2 against the 0.5 mM EDTA pre-treated cell suspensions of *P. mirabilis* Hauser 1885 and *P. mirabilis* BB2000, respectively. The mean relative activity values are plotted; the error bars represent ± standard deviation from three biological replicates. Asterisks indicate significant differences between the relevant exposure times compared to baseline (5°C), * ($p < 0.05$), **($p < 0.01$), ***, ($p < 0.001$), and **** ($p < 0.0001$) using one-way ANOVA and Dunnett's post-test analysis ($n = 3$). (C) The effects of different

concentrations of divalent cations NaCl on the lytic activity of endolysins LysPM1 and LysPM2 . The mean relative activity values are plotted; the error bars represent ± standard deviation from three biological replicates. Asterisks indicate significant differences between the relevant treatment groups compared to baseline (0 mM), * ($p < 0.05$), **($p < 0.01$), ***, ($p < 0.001$), and **** ($p < 0.0001$) using one-way ANOVA and Dunnett's post-test analysis ($n = 3$).

Table S1. Bacterial strains used for host range analysis.

Bacterial strain	Strain source	Reference
***Proteus mirabilis* BB2000**	Reference strain	(Sullivan et al., 2013)
***Proteus mirabilis* ATCC 51286**	Reference strain	ATCC 51286
***Proteus mirabilis* ATCC 35508**	Reference strain	ATCC 35508
***Proteus mirabilis* Hauser 1885**	Reference strain	DSM 788
***Proteus mirabilis* B2**	Clinical isolate	(Stickler et al., 2006)
***Proteus mirabilis* B4**	Clinical isolate	(Jones et al., 2005)
***Proteus mirabilis* RS1**	Clinical isolate	(Milo et al., 2016)
***Proteus mirabilis* RS18**	Clinical isolate	(Pelling et al., 2019)
***Proteus mirabilis* RS28**	Clinical isolate	(Pelling et al., 2019)
***Proteus mirabilis* RS40**	Clinical isolate	(Pelling et al., 2019)
***Proteus mirabilis* RS47**	Clinical isolate	(Pelling et al., 2019)
***Proteus mirabilis* RS50A**	Clinical isolate	(Pelling et al., 2019)
***Proteus mirabilis* Cui0002**	Clinical isolate	–
***Proteus mirabilis* Cui0007**	Clinical isolate	–
***Proteus mirabilis* Cui0013**	Clinical isolate	–
***Proteus mirabilis* Cui0086**	Clinical isolate	–
***Proteus mirabilis* Cui0270**	Clinical isolate	–
***Proteus mirabilis* Cui0321**	Clinical isolate	–
***Proteus mirabilis* Cui0338**	Clinical isolate	–
***Proteus mirabilis* Cui0358**	Clinical isolate	–
***Proteus mirabilis* Cui0364**	Clinical isolate	–
***Proteus vulgaris* UM 266**	Clinical isolate	–
***Proteus penneri* NCTC 12737**	Reference strain	NCTC 12737
***Providencia rettgeri* NCTC 11800**	Reference strain	NCTC 11800
***Providencia stuartii* NCTC 11801**	Reference strain	NCTC 11801
***Morganella morgani* NCTC 235**	Reference strain	NCTC 235

References

Jones, B. V., Mahenthiralingam, E., Sabbuba, N. A., & Stickler, D. J. (2005). Role of swarming in the formation of crystalline *Proteus mirabilis* biofilms on urinary catheters. *J Med Microbiol*, *54*(Pt 9), 807-813. https://doi.org/10.1099/jmm.0.46123-0

Milo, S., Thet, N. T., Liu, D., Nzakizwanayo, J., Jones, B. V., & Jenkins, A. T. A. (2016). An in-situ infection detection sensor coating for urinary catheters. *Biosens Bioelectron*, *81*, 166-172. https://doi.org/10.1016/j.bios.2016.02.059

Pelling, H., Bock, L. J., Nzakizwanayo, J., Wand, M. E., Denham, E. L., MacFarlane, W. M., Sutton, J. M., & Jones, B. V. (2019). De-repression of the smvA efflux system arises in clinical isolates of *Proteus mirabilis* and reduces susceptibility to chlorhexidine and other biocides. *Antimicrob Agents Chemother*, *63*(12). https://doi.org/10.1128/AAC.01535-19

Stickler, D. J., Lear, J. C., Morris, N. S., Macleod, S. M., Downer, A., Cadd, D. H., & Feast, W. J. (2006). Observations on the adherence of *Proteus mirabilis* onto polymer surfaces. *J Appl Microbiol*, *100*(5), 1028-1033. https://doi.org/10.1111/j.1365-2672.2006.02840.x

Sullivan, N. L., Septer, A. N., Fields, A. T., Wenren, L. M., & Gibbs, K. A. (2013). The Complete Genome Sequence of *Proteus mirabilis* Strain BB2000 Reveals Differences from the *P. mirabilis* Reference Strain. *Genome Announcements*, *1*(5). https://doi.org/10.1128/genomea.00024-13

Table S2. Protein sequences of endolysins LysPM1 and LysPM2.

Name	Source	Sequence	Length, aa	GenBank accession
LysPM1	Proteus phage vB_PmiP-Homer	MNEYFKRKEFACKCGCGTSTVDAELLQVITDVREHFGKPVIITSGHRCFKHNTNVGGARNSMHLTGKAADIKVKGVDPADVADYLETKYPEQYGIGRYAGRFTHIDVREYKARWRG	116	–
LysPM2	Proteus phage vB_PmiS_PM-CJR	MIKWLEEAKKEIGVSEHTIEGSKAVDQMWRDSKMSGLVGTAKTVPWCAGFVNAMLERSGICSTRSDSSKSYIDYGVKLSEPKYGCIVVFSRSGGGHVGFCVGKTKDGNLLILGGNQSDAVNIKSFSTDRAIAYRYPDIGFIDNRPLPIGEAGMSIKES	158	QYC51792

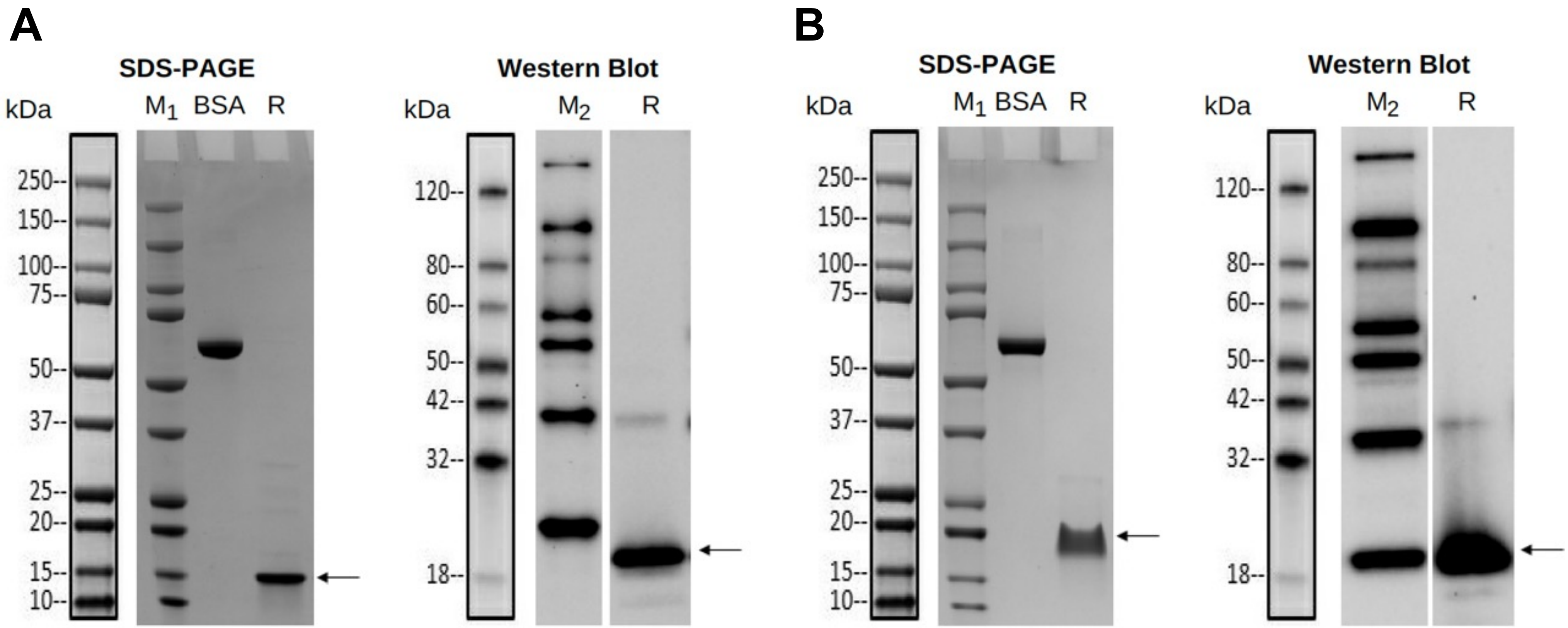

Figure S1. The SDS-PAGE and Western blot (with mouse anti-His-tag antibodies) results provided by GenScript. (A) LysPM1 and (B) LysPM2 endolysin. In SDS-PAGE: M_1 – protein marker , BSA – bovine serum albumin (2 μg), R – purified endolysin sample (reducing conditions). In Western blot: M_2 – protein marker , R – purified endolysin sample (reducing conditions). Arrows indicate the presence of a band of the expected molecular weight.

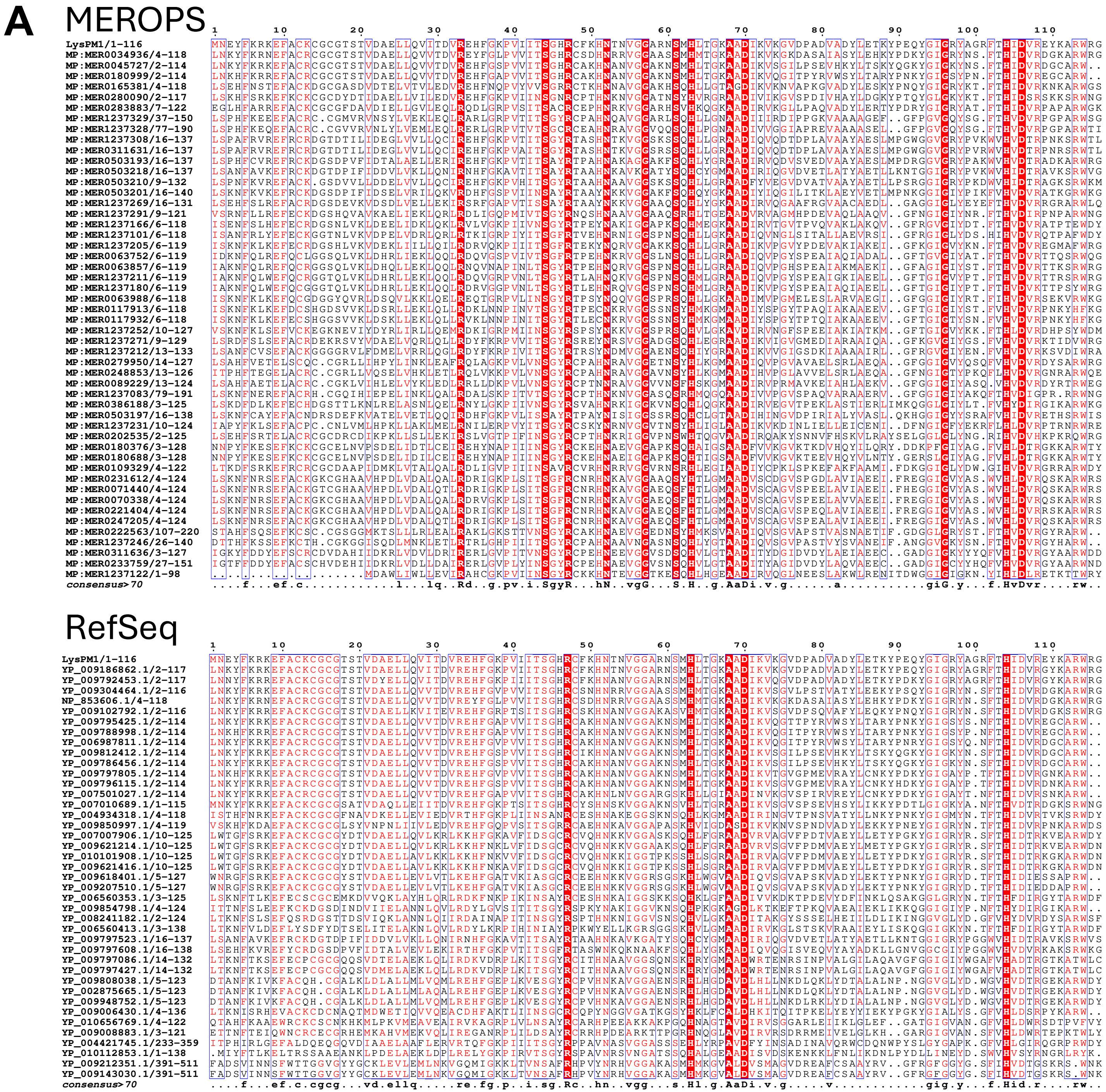
A
MEROPS
RefSeq

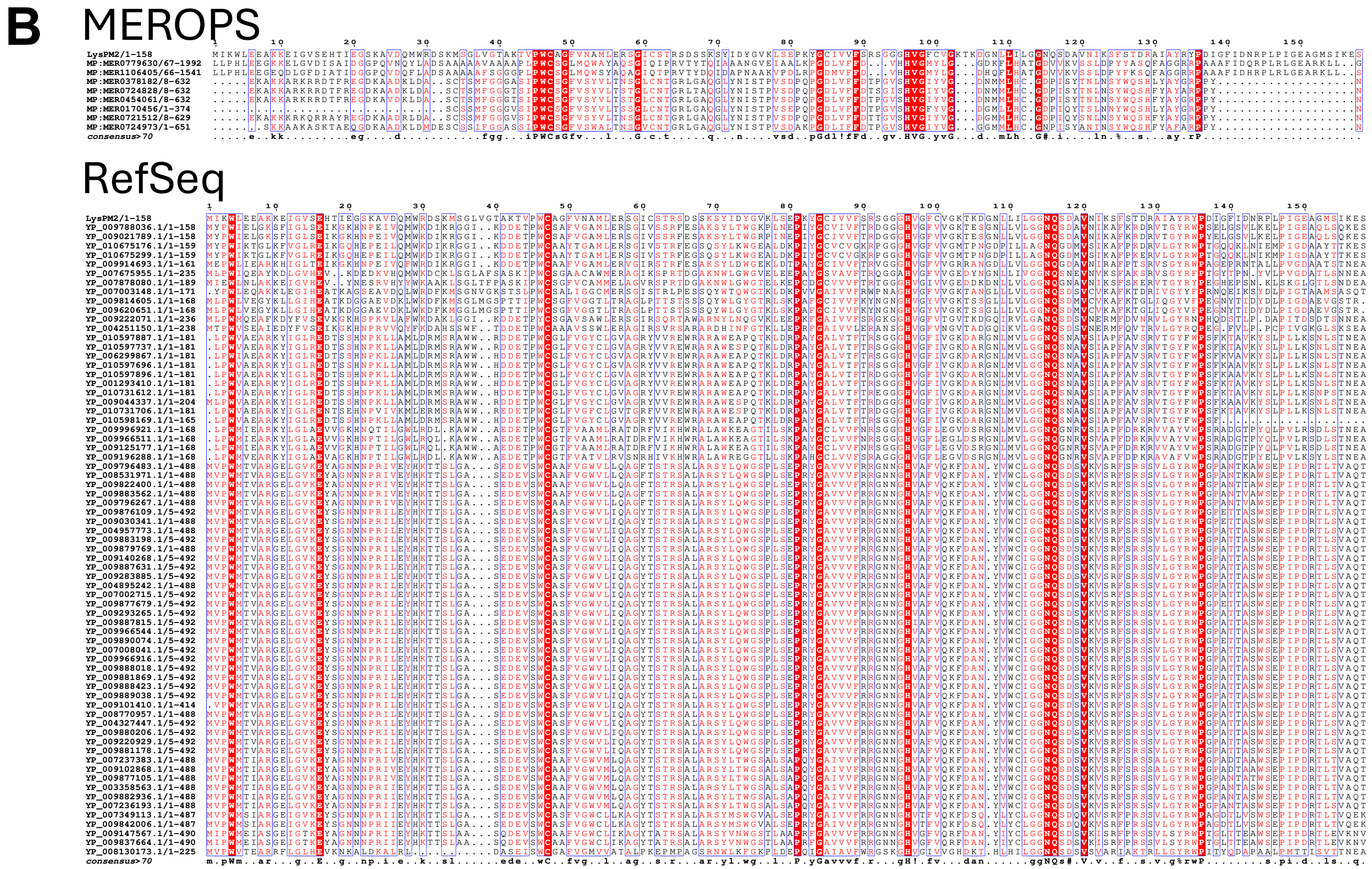

Figure S2. Multiple sequence alignments of endolysins LysPM1 (A) and LysPM2 (B) with BlastP hits from MEROPS and RefSeq databases produced by MAFFT, edited in Jalview, and visualised by ESPript.

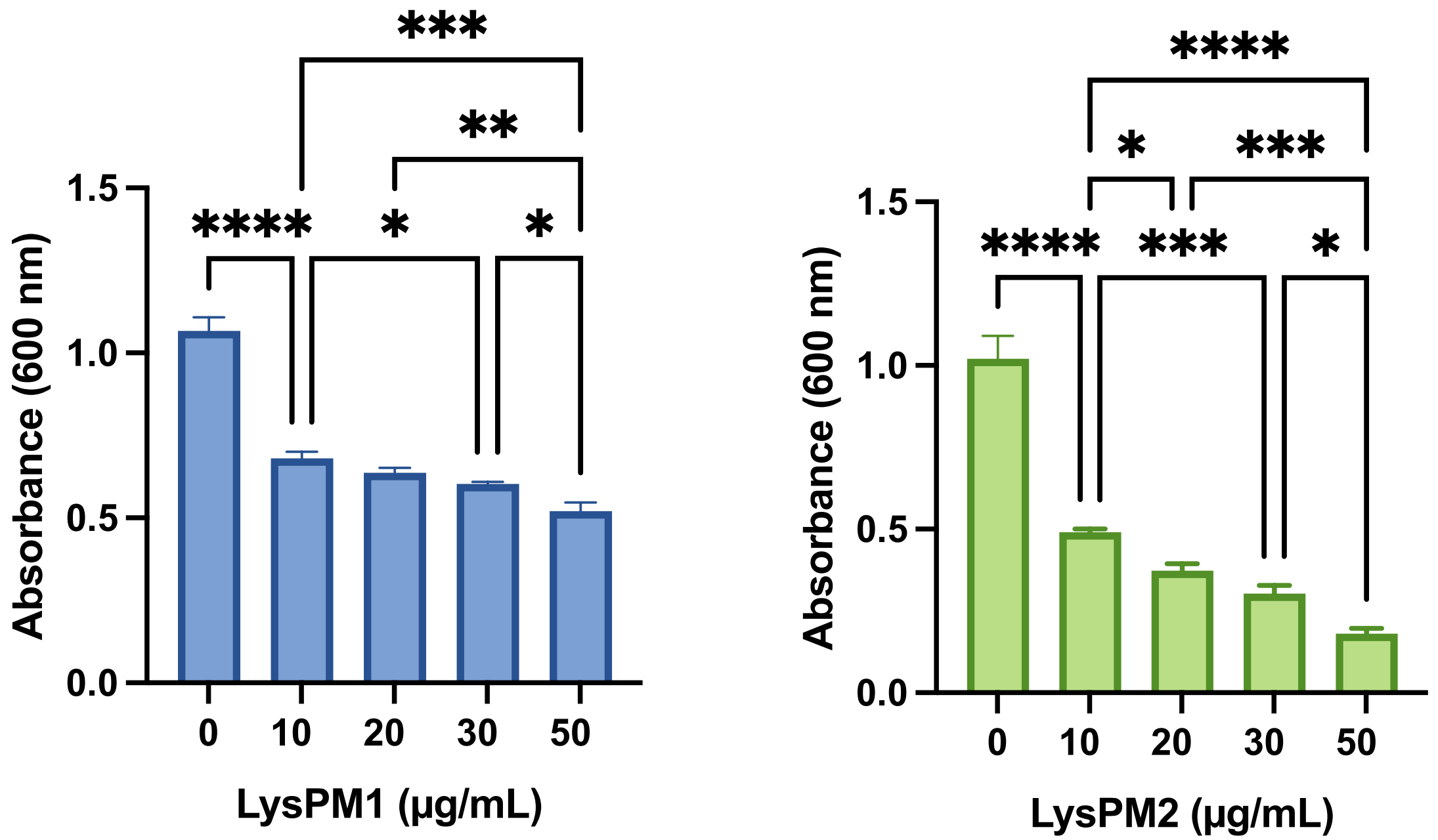
***
**
****
*
*
1.5
1.0
0.5
0.0
Absorbance (600 nm)
0
10
20
30
50
LysPM1 (µg/mL)
****
*
***
****
***
*
1.5
1.0
0.5
0.0
Absorbance (600 nm)
0
10
20
30
50
LysPM2 (µg/mL)

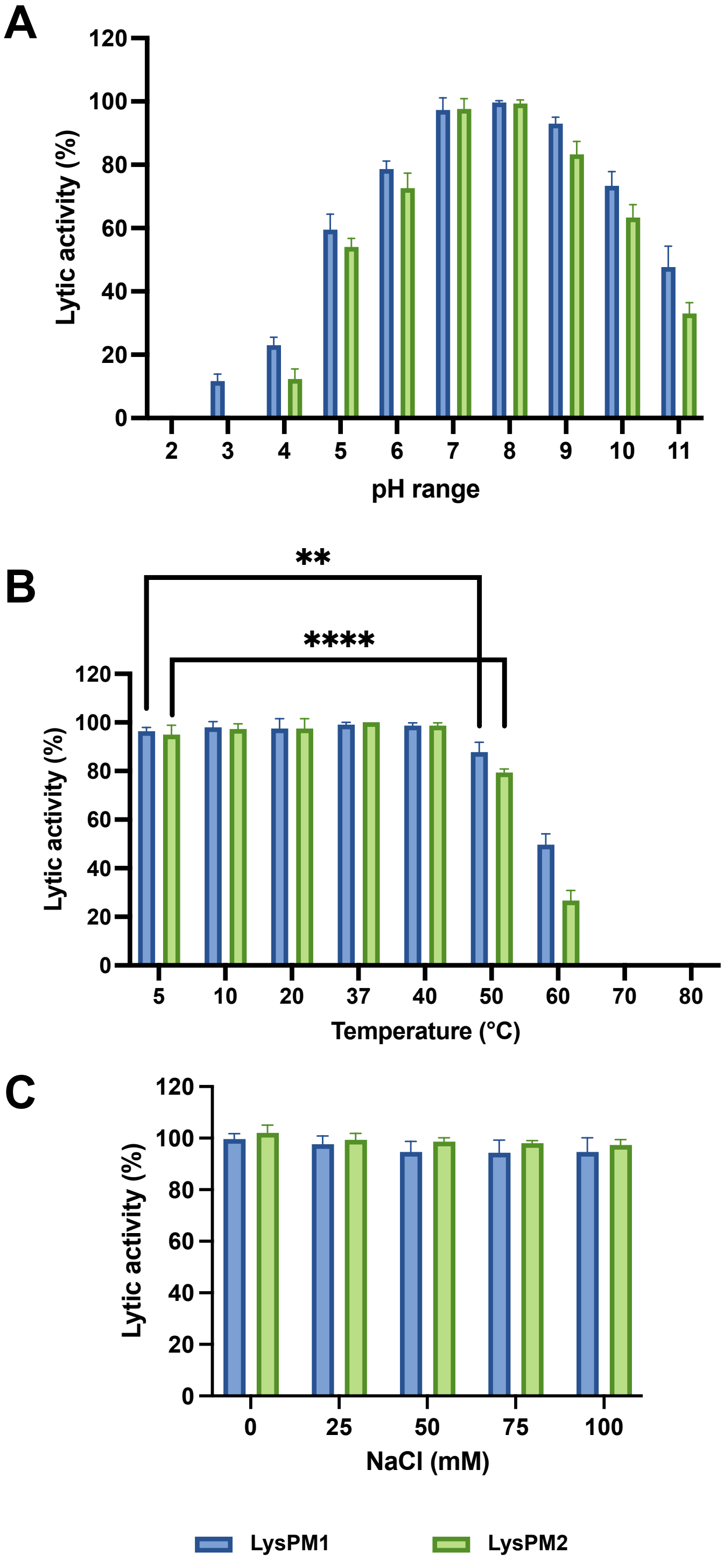
A
120
100
80
60
40
20
0
Lytic activity (%)
2
3
4
5
6
7
8
9
10
11
pH range
B
**
****
120
100
80
60
40
20
0
Lytic activity (%)
5
10
20
37
40
50
60
70
80
Temperature (°C)
C
120
100
80
60
40
20
0
Lytic activity (%)
0
25
50
75
100
NaCl (mM)
LysPM1
LysPM2
